# Supplementary figures and images for: Genomics-enabled analysis of the emergent disease cotton bacterial blight
Source: PLoS Genet. 2017 Sep 14;13(9):e1007003. doi: 10.1371/journal.pgen.1007003 (PMC5614658; doi:10.1371/journal.pgen.1007003)

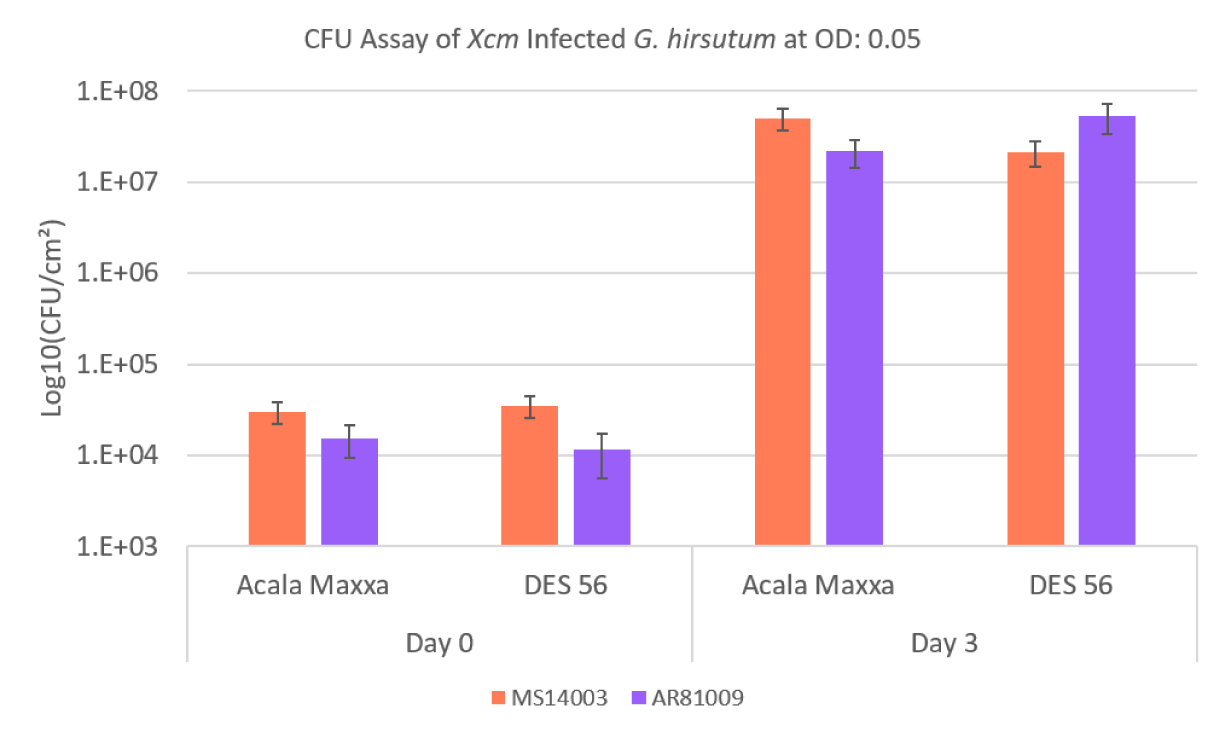

Supplement: S3 Fig — G. hirsutum varieties were inoculated with Xcm at an OD600: 0.05. Tissue was collected at day 0 and day 3 and processed as described in materials and methods. (TIF) [file pgen.1007003.s007.tif]

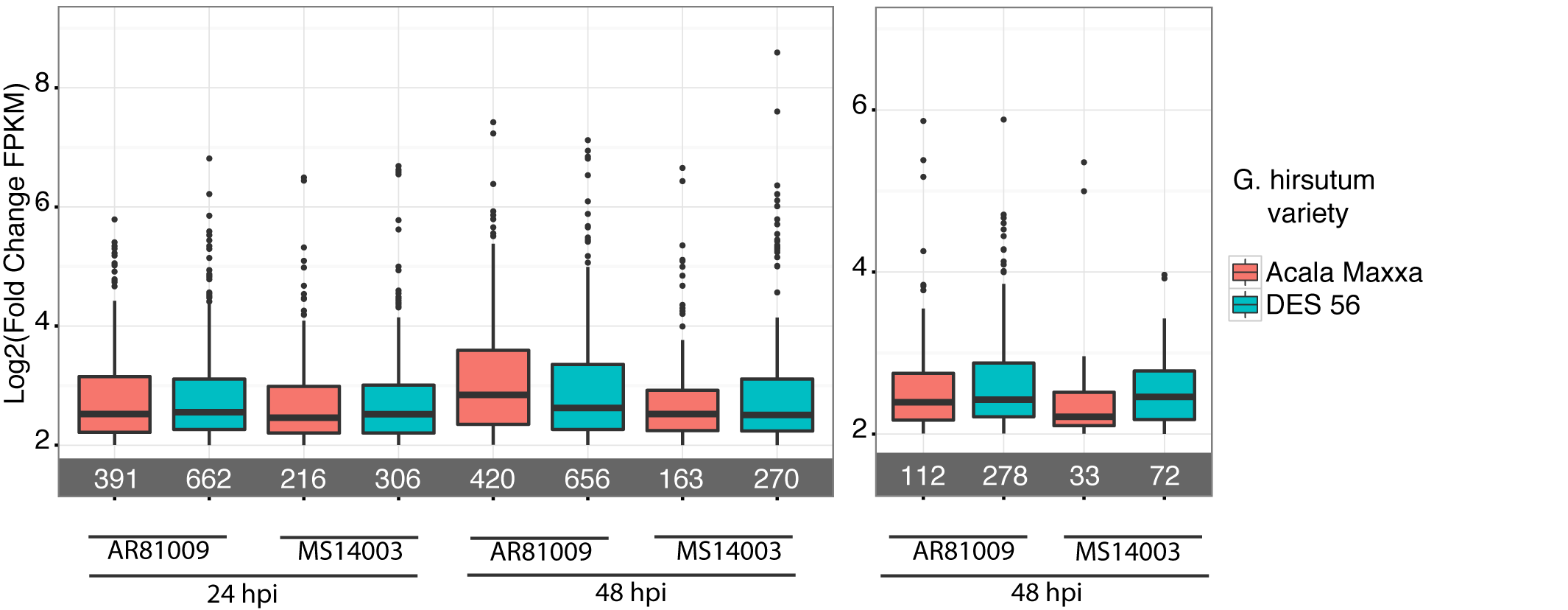

Supplement: S4 Fig — A) All significantly upregulated genes with a Log2 fold change of 2 B) All significantly upregulated genes (p ≤ 0.05) with a Log2 (fold change in FPKM) ≥ 2 that are unique to each cultivar/Xcm disease interaction in G. hirsutum. Numbers in grey bar indicate the total number of genes for each condition. (TIF) [file pgen.1007003.s008.tif]

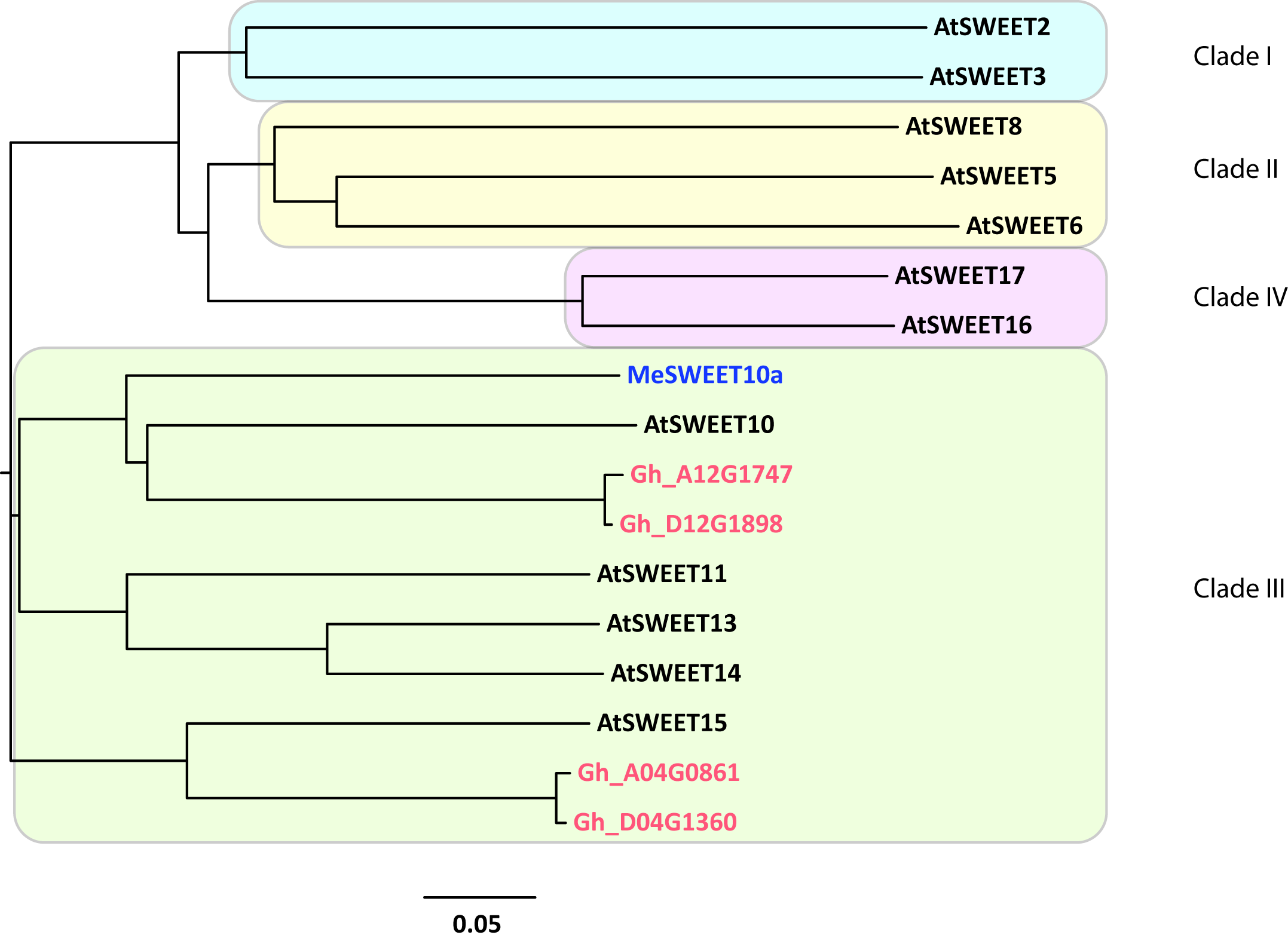

Supplement: S5 Fig — Four predicted G. hirsutum SWEET genes are compared to classified A. thaliana SWEET genes and the MeSWEET10a M. esculenta susceptibility gene. A protein alignment and phylogenetic tree was generated by Clustal Omega, and the tree was visualized using Figtree v1.4.2. (TIF) [file pgen.1007003.s009.tif]
